# Longitudinal Analysis of Electronic Health Records Reveals Medical Conditions Associated with Subsequent Alzheimer's Disease Development

**DOI:** 10.1002/alz70860_106739

**Published:** 2025-12-23

**Authors:** Xue Zhong, Gengjie Jia, Zhijun Yin, Kerou Chen, Bingshan Li, Andrey Rzhetsky, Nancy J Cox

**Affiliations:** ^1^ Vanderbilt Genetics Institute, Vanderbilt University Medical Center, Nashville, TN, USA; ^2^ University of Chicago, Chicago, IL, USA; ^3^ Vanderbilt University Medical Center, Nashville, TN, USA; ^4^ Vanderbilt University, Nashville, TN, USA; ^5^ Vanderbilt Genetics Institute, Nashville, TN, USA

## Abstract

**Background:**

Managing certain health conditions may promote brain health and reduce the risk of Alzheimer's disease (AD). It is projected that delayed onset of AD by just five years could reduce the population‐level incidence by 50%.

**Method:**

In this study, we used electronic health records (EHRs) from two independent databases with more than 153 million individuals to systematically identify medical conditions that are associated with subsequent development of AD. By tracking the EHRs over 10 years before receiving AD diagnosis and comparing the prevalence of the preceding conditions between AD cases and age‐ and gender‐matched controls, we discovered and validated medical conditions that occurred more frequently in those who later developed AD. We further assessed the enriched phenotypes for connection to AD genetics in two large‐scale biobanks (BioVU and UK Biobank).

**Result:**

In the MarketScan database, a claim‐based national‐level database with EHRs from over 150 million individuals, we identified 43,508 AD cases and 409,667 matched controls; in the Vanderbilt University Medical Center (VUMC) hospital‐based database, which contains de‐identified EHRs from over 3 million patients, we identified 1,394 AD cases and 11,624 matched controls. Our enrichment analysis reveals more than 70 medical conditions that are consistently replicated across independent EHR databases, including multiple known risk factors of AD (e.g., hypertension, hypercholesterolemia, diabetes, depression, and sleep disorders) and less reported associations such as hypothyroidism. Several conditions (e.g., psychosis, diabetes, vitamin B‐complex deficiencies etc.) align with several prescribed drugs (e.g. antipsychotics, anti‐diabetic drugs, vitamins and supplements) newly reported for positive association with AD. We found a subset of the enriched phenotypes were supported by AD genetics, showing associations with AD risk variants or genetic risk score.

**Conclusion:**

Our analyses of longitudinal EHRs from millions of individuals robustly identified health conditions associated with higher incidence rate of AD, calling attention to better management of these health conditions to promote brain health and reduce AD risk.

